# Beyond just “flattening the curve”: Optimal control of epidemics with purely non-pharmaceutical interventions

**DOI:** 10.1186/s13362-020-00091-3

**Published:** 2020-08-18

**Authors:** Markus Kantner, Thomas Koprucki

**Affiliations:** Weierstrass Institute for Applied Analysis and Stochastics (WIAS), Mohrenstr. 39, Berlin, 10117 Germany

**Keywords:** 92D30, 37N25, 37N40, 93C10, 49N90, 34B15, Mathematical epidemiology, Optimal control, Non-pharmaceutical interventions, Reproduction number, Dynamical systems, COVID-19, SARS-CoV2

## Abstract

When effective medical treatment and vaccination are not available, non-pharmaceutical interventions such as social distancing, home quarantine and far-reaching shutdown of public life are the only available strategies to prevent the spread of epidemics. Based on an extended SEIR (susceptible-exposed-infectious-recovered) model and continuous-time optimal control theory, we compute the optimal non-pharmaceutical intervention strategy for the case that a vaccine is never found and complete containment (eradication of the epidemic) is impossible. In this case, the optimal control must meet competing requirements: First, the minimization of disease-related deaths, and, second, the establishment of a sufficient degree of natural immunity at the end of the measures, in order to exclude a second wave. Moreover, the socio-economic costs of the intervention shall be kept at a minimum. The numerically computed optimal control strategy is a single-intervention scenario that goes beyond heuristically motivated interventions and simple “flattening of the curve”. Careful analysis of the computed control strategy reveals, however, that the obtained solution is in fact a tightrope walk close to the stability boundary of the system, where socio-economic costs and the risk of a new outbreak must be constantly balanced against one another. The model system is calibrated to reproduce the initial exponential growth phase of the COVID-19 pandemic in Germany.

## Introduction

Preventing the spread of new diseases, to which there is no immunity in the population, is a huge problem, since there are often neither vaccines nor other effective medical treatments available in the early stages. In this case, non-pharmaceutical interventions (NPIs) such as intensive hand hygiene, home quarantine and measures of social distancing, e.g. closure of schools, universities and shops, prohibition of mass events up to curfew and shutdown of entire territories, are the only available measures. The NPIs are aimed at “flattening the curve”, i.e., a reduction of the transmission rate in order to break the exponential growth of the epidemic.

In the case of the currently spreading COVID-19 pandemic caused by the new SARS-CoV2 coronavirus [1, 2], the fundamental concern of the mitigation measures is not to exceed the available number of intensive care unit (ICU) beds, in particular for respiratory support or extracorporeal membrane oxygenation, in order to prevent actually avoidable deaths [3]. Since the outbreak of the epidemic, a large number of simulation studies have been conducted using mathematical models to assess the efficacy of different NPIs and to estimate the corresponding demands on the health care system [4–12]. Moreover, mathematical models are employed to deduce important epidemiological parameters [13–15] and to evaluate the effect of particular measures from empirical data [16, 17].

The vast majority of research papers on the control of COVID-19 examines the impact of rather simple intervention schemes such as bang-bang control or cascaded on-off (i.e., repeated lockdown and release) strategies [12, 18–20]. Instead, however, intervention strategies derived from continuous-time optimal control theory [21] following a variational principle are actually preferable. There is a large number of studies on the application of optimal control theory following Pontryagin’s maximum principle [22] in mathematical epidemiology, see Refs. [23–27] and references therein. The by far largest part of these works deals with optimal control of epidemics through vaccination and immunization [28–31], medical treatment [32, 33] and combinations thereof [34–39]. Significantly fewer papers are concerned with the optimal control of transmission dynamics and the mitigation of epidemics through social distancing measures. The paper by Behncke [25] studies the optimal control of transmission dynamics via optimally steered health-promotion campaigns and seems to be one of the first works devoted to this problem. During the current COVID-19 pandemic, the control of the disease by NPIs has moved into the focus of attention and a number of recent papers are devoted to this problem. Djidjou-Demasse et al. [40] investigated the optimal control of the epidemic via social distancing and lockdown measures until a vaccine becomes available. They propose to delay the peak of the epidemic by increasingly strict interventions and finally to relax the measures in such a way that a significant burden on the health care system only occurs when the availability of a vaccine is already expected. A similar problem has been considered by Perkins and España [41], who studied the optimal implementation of NPIs under the assumption that an effective vaccine would become available in about one year after the outbreak of the epidemic. The paper by Kruse and Strack [42] is devoted to the analysis of the optimal timing of social distancing measures under the constraint that the overall (temporal) budget for NPIs is limited. Ketcheson [43] presented a detailed analysis for optimal transmission control in a SIR (susceptible-infected-recovered) epidemic model with the aim of achieving a stable equilibrium (“herd immunity”) within a fixed finite time interval while simultaneously avoiding hospital overflow. A similar problem (including a simple state-dependent mortality rate) was studied by Alvarez et al. [44], who focussed on minimizing the lockdown costs and included further economic aspects such as the assumed value of statistical life. An extension of the optimal transmission control problem to an age-structured model has been presented by Bonnans and Gianatti [45], who proposed a different temporal course of the contact reduction for the high and low risk sub-populations. Köhler et al. [46] have applied model predictive control to social distancing measures with the objective of minimizing the fatalities over a fixed period of time of two years. Next to adaptive feedback strategies for iterative loosening of the social distancing policies after an initial lockdown, the authors also examined the possibility of eradicating the virus. All of these papers on optimal control deal with deterministic epidemiological models, in particular the basic SIR model [25, 42–44, 47] or various extended SEIR-type models [40, 41, 46]. We remark that this survey on optimal control of COVID-19 is not exhaustive.

The objective of this paper is the investigation of the optimal control of epidemics in the (hopefully unlikely) case in which an effective vaccine is impossible or never found and the epidemic must be controlled with purely non-pharmaceutical measures. Furthermore, we exclude the possibility of complete containment (“eradication of the virus”). Then, optimal control must pursue competing objectives: On the one hand, the number of disease-related deaths shall be minimized by strictly avoiding an overload of the intensive care treatment capacities. On the other hand, however, sufficient natural immunity must be established in the population in the long run to prevent a second outbreak of the epidemic (“herd immunity”). Moreover, the socio-economic costs of the intervention shall be kept at a minimum. We compute the optimal solution to this problem by applying Pontryagin’s Maximum Principle to an extended SEIR-type model tailored to specific aspects of COVID-19. Our main result is the optimal time course of the mean contact reduction (and the corresponding time-dependent effective reproduction number) that serves as a guideline on how to optimally enter and finally exit the lockdown. The corresponding NPI policy is a single-intervention scenario that can be divided into three distinct phases: (1) a strict initial lockdown, (2) a long lasting period (“critical period”) during which the number of active cases is kept approximately constant and (3) a moderate tightening of the measures towards the end of the intervention. We present a detailed analysis of the numerically computed result and develop an analytical understanding of its distinct features. Moreover, we show that our numerically computed optimal control obeys two fundamental stability criteria, which impose an upper limit on the transmission rate and its rate of change on the way out of the initial lockdown. The precise structure of the optimal control (i.e., three phases of the intervention) obtained in this paper differs from the results described in similar works [42–44]. After the initial submission of this paper, the preprint by Charpentier et al. [48] appeared, who studied a similar optimization problem on the basis of an extended SIR-type model with parameters adjusted to the COVID-19 pandemic in France. Their independently obtained results are comparable to those presented in this paper, which demonstrates the robustness of the obtained optimal intervention strategy with respect to model and parameter variations.

The mathematical model for the progression of the epidemic and the estimation of the demand for intensive care resources is described in Sect. 2. The optimal control problem is derived in Sect. 3 and the results are described in Sect. 4. We close with a critical discussion of our findings in Sect. 5. The model has been calibrated to reproduce the exponential growth phase of the COVID-19 pandemic in Germany. Details on the parameter adjustment are described in the Appendix.

## Modeling of disease spreading and demand for intensive care units

Mathematical modeling of the spread of epidemics is an indispensable tool to project the outcome of an epidemic, estimate important epidemiological parameters and to make predictions for different intervention scenarios. Compartment models [49–51], where the population is divided into different macroscopic sub-populations, such as *susceptible*, *infectious*, *recovered* etc., are a simple but effective tool to model the progression of epidemics. In contrast to complex (but more realistic) stochastic agent-based models [52, 53], deterministic mean-field models are limited to the description of the average infection dynamics in macroscopic (sub-)populations, but allow for fast parameter scans and a straightforward application of continuous-time optimal control theory [21].

### Model equations

In this paper, an extended SEIR model, similar to that proposed by Neher et al. [54, 55], is used to model the spread of an epidemic and to estimate the number of patients in a critical state that require intensive care. Similar models are described in Refs. [14, 46, 56]. For the sake of simplicity, vital dynamics (except for disease-related deaths), seasonality effects [57], dispersion of transmission [58] and any effects caused by population heterogeneity (different age and risk groups) are neglected. The total population is divided into distinct compartments: susceptible *S*, exposed *E*, infectious *I*, hospitalized *H* (severely ill), critical *C*, recovered *R* (i.e., immune) and deceased *D*. The model equations read

1a $$\begin{aligned} &\dot{S} =-\beta u (t )\frac{IS}{N}, \end{aligned}$$

1b $$\begin{aligned} &\dot{E} =\beta u (t )\frac{IS}{N}-\gamma _{l}E, \end{aligned}$$

1c $$\begin{aligned} &\dot{I} =\gamma _{l}E-\gamma _{i}I, \end{aligned}$$

1d $$\begin{aligned} &\dot{H} = (1-m )\gamma _{i}I+ \bigl(1-f (C/C_{0} ) \bigr)\gamma _{c}C-\gamma _{h}H, \end{aligned}$$

1e $$\begin{aligned} &\dot{C} =c\gamma _{h}H-\gamma _{c}C, \end{aligned}$$

1f $$\begin{aligned} &\dot{R} =m\gamma _{i}I+ (1-c )\gamma _{h}H, \end{aligned}$$

1g $$\begin{aligned} &\dot{D} =f (C/C_{0} )\gamma _{c}C. \end{aligned}$$

The group of initially healthy and not yet infected (susceptible, *S*) is vulnerable to infection through contact with infectious (*I*), who may transmit the disease to the susceptible population. The infection probability is determined by the transmission rate *β*, and the share of the susceptible and infectious population on the total (living) population $N=N(t)$, which is given as

2 $$ N=S+E+I+H+C+R. $$

**Figure 1:**
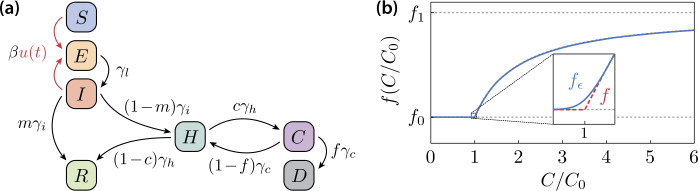
(**a**) Schematic illustration of the compartmental epidemic model (1a)–(1g). The function $u (t )$ describes a modification of the transmission dynamics due to NPIs. (**b**) State-dependent mortality rate *f* as a function of the number of patients in a critical state requiring intensive care. The mortality rate grows rapidly if the number of critical patients exceeds the number of available ICUs $C_{0}$. Inset: The solid line is the regularized mortality rate (4b) that is used in the computations throughout the paper

The newly infected (exposed, *E*) become infectious themselves only after a latency period $\gamma _{l}^{-1}$ (which must not be confused with the incubation time). The infectious either recover or turn severely ill after an average period $\gamma _{i}^{-1}$. Severely ill (*H*) can either deteriorate into a critical state (*C*) or recover after a period $\gamma _{h}^{-1}$. The recovered population (*R*) is assumed to be immune against new infections. Patients in a critical state either stabilize to the severely ill state or die from the disease on a time scale $\gamma _{c}^{-1}$. The disease-related deaths reduce the size of the population

3 $$ \dot{N}=-\dot{D}, $$

such that, assuming initially $D (0 )=0$, it holds $N (t )=N (0 )-D (t )$. Moreover, *m* is the share of infectious that are asymptomatic or have at most mild symptoms, *c* is the fraction of severely ill that become critical and *f* is the fraction of critically ill that are going to die from the disease. Finally, the time-dependent function $u (t )$ describes a modification of the transmission rate (mean contact reduction) due to NPIs. Here, $u=1$ means no intervention, and $u=0$ corresponds to the extreme case of total isolation of the whole population. The model system is illustrated in Fig. 1(a). A rescaled version of the dynamical system (1a)–(1g), where the sub-populations are considered in terms of their relative share of the initial population $N (0 )$, is given in Eq. (23a)–(23g) in the Appendix D.

### State-dependent fatality rate

The disease-related mortality grows tremendously as soon as the number of critically ill exceeds the capacity limit $C_{0}$ of the health care system (number of available ICUs). This is modeled by a state-dependent average fatality rate

4a $$ f=f \biggl(\frac{C}{C_{0}} \biggr)= \textstyle\begin{cases} f_{0} & \text{for }C\leq C_{0}, \\ f_{1}-\frac{C_{0}}{C} (f_{1}-f_{0} ) & \text{for }C>C_{0}. \end{cases} $$

As long as every critical patient can be served with an ICU ($C\leq C_{0}$), the fatality rate is a constant $f=f_{0}$. As soon as the ICU resources are exceeded, an increasing fraction of the critical patients dies with a higher rate $f_{1}>f_{0}$, which on average results in the state-dependent fatality rate (4a). Here, $f_{1}=2f_{0}$ is assumed. In the following, the regularization

4b $$ f (x )\to f_{\epsilon } (x )=f_{0}+ \frac{\epsilon }{x+1.1\epsilon }\log { \biggl(1+\exp { \biggl( \frac{x-1}{\epsilon } \biggr)} \biggr)} (f_{1}-f_{0} ) $$

with $0<\epsilon \ll 1$, of Eq. (4a) is used, in order to avoid problems due to the non-differentiability at $C=C_{0}$. The function $f (C/C_{0} )$ is plotted in Fig. 1(b).

### Basic and effective reproduction number

The basic reproduction number [59]

5 $$ \mathcal{R}_{0}=\beta /\gamma _{i} $$

can be thought of as the expected number of cases (without intervention, $u=1$) that is directly generated by one case in a population where all individuals are susceptible to infection. The effective reproduction number

6 $$ \mathcal{R}_{\text{eff}} (t )=\mathcal{R}_{0}u (t )S (t )/N (t ) $$

depends on time and includes the impact of intervention measures.

### Numerical results for the uncontrolled epidemic (COVID-19 in Germany)

**Figure 2:**
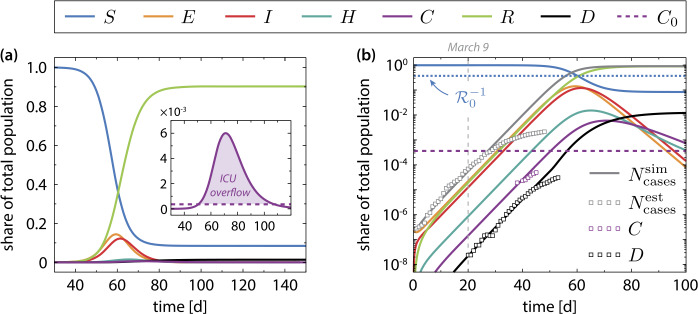
(**a**) Evolution of the epidemic without interventions ($u=1$). The number of available ICUs was set to $C_{0}=30\text{,}000$. The inset shows the overflow in ICU demand, which leads during a period of about 57 days to an increased mortality rate according to Eq. (4a)–(4b). (**b**) Same as in (**a**) but on a logarithmic scale. The markers indicate the estimated number of cumulative cases (see Appendix C) and the reported numbers for ICU demand and deaths during the early phase of the COVID-19 pandemic in Germany. The first disease-related fatalities were reported on March 9, 2020 (day number 20 in the simulation). Social distancing measures, which came into force nationwide in mid-March [16], have flattened the initial exponential growth

Figure 2 shows the progression of an uncontrolled epidemic starting from an initially small fraction of exposed population. The initial conditions are listed in Appendix D. The parameters are adjusted (see Appendix C) to reproduce the initial exponential growth phase of the COVID-19 disease in Germany (late February – mid March 2020) and are summarized in Table 1. The numerical solution was obtained by a 4th order Runge–Kutta method. Without intervention, the peak number of simultaneously active cases is about 23 million and the peak number of patients in a critical state exceeds the number of ICUs by a factor of about $C_{\text{max}}/C_{0}\approx 16.7$, see inset of Fig. 2(a). The simulated value $C_{\text{max}}\approx 5.0\times 10^{5}$ is in very good agreement with the projection by Khailaie et al. [14]. Due to the increased fatality in the period with ICU overflow, see Eq. (4a)–(4b), the epidemic terminates with a very high number of deaths $D (T )\approx 1.0\times 10^{6}$, which is in line with previous studies [11].

**Table 1:** List of parameters used in the simulations. See Appendix C for details

Symbol	Value	Description
$\mathcal{R}_{0}$	2.7	basic reproduction number
*N*(0)	83 × 10^6^	initial population size
$\gamma _{l}^{-1}$	2.6d	average latency time between exposure and infectious period
$\gamma _{i}^{-1}$	2.35d	average infectious period before recovery or hospitalization
$\gamma _{h}^{-1}$	4.0d	average period before severely ill patients turn critical or recover
$\gamma _{c}^{-1}$	7.5d	average period before critical patients recover or die
*β*	$(1.15 \text{d} )^{-1}$	transmission rate
*m*	0.92	fraction of infected with at most mild symptoms
*c*	0.27	fraction of hospitalized patients that turn critical
*f*	see Eq. (4a)–(4b)	fraction of critical patients that turn fatal
$f_{0}$	0.31	mortality of a critical patient with ICU
$f_{1}$	2 $f_{0}=0.62$	mortality of a critical patient without ICU
$C_{0}$	variable	number of ICUs/ max. number of simultaneously critical cases
*T*	$10\times T_{\text{crit}}$	final time of the simulation, for $T_{\text{crit}}$ see Eq. (17)

## Optimal control

**Figure 3:**
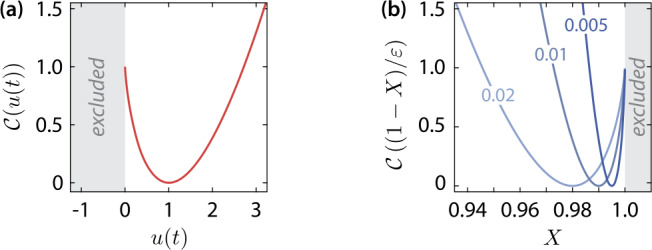
Plot of the cost functions for (**a**) minimal intermediate costs and (**b**) the enforcement of herd immunity at the end of the intervention for different values of *ε*. We use the short notation $X=\mathcal{R}_{0}S (T )/N (T )$. The shaded region corresponds to unstable terminal states

In the scenario outlined in Sect. 1, where an effective vaccine is never found, the optimal transmission control due to NPIs is required (i) to avoid ICU overflow (more patients in a critical state than available ICUs) but at the same time (ii) exclude a second wave of the epidemic after the end of the measures. The optimal solution is computed by minimizing the index functional

7a $$ \mathcal{J} [u ]=\varphi \bigl(\mathbf{x} (T ) \bigr)+ \int _{0}^{T}\mathrm{d}t \, \mathcal{C} \bigl(u (t ) \bigr), $$

where

7b $$ \varphi \bigl(\mathbf{x} (T ) \bigr)=\mathcal{P} D (T )+\mathcal{C} \biggl( \frac{1}{\varepsilon } \biggl(1- \mathcal{R}_{0}\frac{S (T )}{N (T )} \biggr) \biggr) $$

is the terminal cost function. The first term in Eq. (7b) describes the number of disease-related deaths $D (T )$ at the end of the epidemic, which should be minimized. As the increment of the disease-related deaths depends on the state-dependent fatality rate, see Eq. (1g), this condition implies that the ICU capacities must not be exceeded. The second term in Eq. (7b) controls the size of the of susceptible population $S (T )$ at the end of the epidemic. In order to approach a stable, disease-free stationary state (“herd immunity”), the share of susceptibles on the total population must be less than $\mathcal{R}_{0}^{-1}$ at the end of the intervention, see Appendix A. The term in Eq. (7b) enforces a final state slightly below the stability boundary (just in the stable regime), where $0<\varepsilon \ll 1$ is a small parameter. We use $\varepsilon =10^{-2}$ in the numerical simulations throughout this paper. The function

8 $$ \mathcal{C} (x )=x\log { (x )}-x+1 $$

is convex on the whole domain $x\in [0,\infty )$. It appears also in the last term of Eq. (7a) as an intermediate cost function, which provides an abstract measure for the total socio-economic costs caused by the intervention. The term is minimal and zero if no intervention is applied $\mathcal{C} (1 )=\mathcal{C}' (1 )=0$, see Fig. 3. The advantage of using (8) over the commonly used quadratic cost functions is that “unphysical” negative values of *u* are a priori excluded. The control parameter $\mathcal{P}$ balances between the competing objectives of minimal disease-related deaths (first term), while attaining at the same time a minimum number of cases to enforce $S (T )$ slightly below the stability boundary (second term). Ramping up $\mathcal{P}$ puts an increasing emphasis on minimizing the disease-related deaths. The time interval $[0,T]$ of the simulation is chosen sufficiently large, such that the results are practically independent from the chosen final time *T*, see Table 1.

From the augmented index functional [21]

$$ \bar{\mathcal{J}} [u ]=\varphi \bigl(\mathbf{x} (T ) \bigr)+ \int _{0}^{T}\mathrm{d}t \, \bigl(\mathcal{C} \bigl(u (t ) \bigr)+\boldsymbol{\lambda } (t )\cdot \bigl(\mathbf{F} \bigl( \mathbf{x} (t ),u (t ) \bigr)-\dot{\mathbf{x}} (t ) \bigr) \bigr), $$

where $\mathbf{x}= (S,E,I,H,C,R,D )$ is the state vector, $\dot{\mathbf{x}}=\mathbf{F} (\mathbf{x},u )$ is the dynamical system (1a)–(1g) and $\boldsymbol{\lambda } (t )$ is a vector of time-dependent Lagrange multipliers (also denoted as *co-state variables*) $\boldsymbol{\lambda }= (\lambda _{S},\lambda _{E},\lambda _{I}, \lambda _{H},\lambda _{C},\lambda _{R},\lambda _{D} )$, one obtains the Hamiltonian function

9 $$ \mathcal{H} (\mathbf{x},u,\boldsymbol{\lambda } )= \mathcal{C} (u )+\boldsymbol{ \lambda }\cdot \mathbf{F} (\mathbf{x},u ). $$

Following Pontryagin’s maximum principle [21, 22], the optimality condition reads

10 $$ \frac{\partial \mathcal{H}}{\partial u}=0 \quad\Leftrightarrow\quad u=\exp { \biggl(\beta (\lambda _{S}-\lambda _{E} ) \frac{IS}{N} \biggr)}. $$

Finally, the co-state equations and the final time conditions are obtained as

11 $$\begin{aligned} &\dot{\boldsymbol{\lambda }} (t ) =-\nabla _{\mathbf{x}} \mathcal{H}, \end{aligned}$$

12 $$\begin{aligned} &\boldsymbol{\lambda } (T ) =\nabla _{\mathbf{x}}\left . \varphi ( \mathbf{x} )\right \vert _{T}. \end{aligned}$$

Together with the initial conditions $\mathbf{x} (0 )$, the system (1a)–(1g), (11)–(12) represents a nonlinear two-point boundary value problem. The full set of equations is given in Appendix D. Numerical solutions are obtained by using Matlab’s built-in routine bvp4c [60] in combination with an analytic Jacobian matrix and a step-size adaptive homotopy method, where the control parameter $\mathcal{P}$ is gradually ramped up while always using the result of the previous step as initialization. The procedure is initiated from the numerical solution of the initial value problem (1a)–(1g) without interventions, see Fig. 2.

## Results

### Structure of the optimal intervention strategy

**Figure 4:**
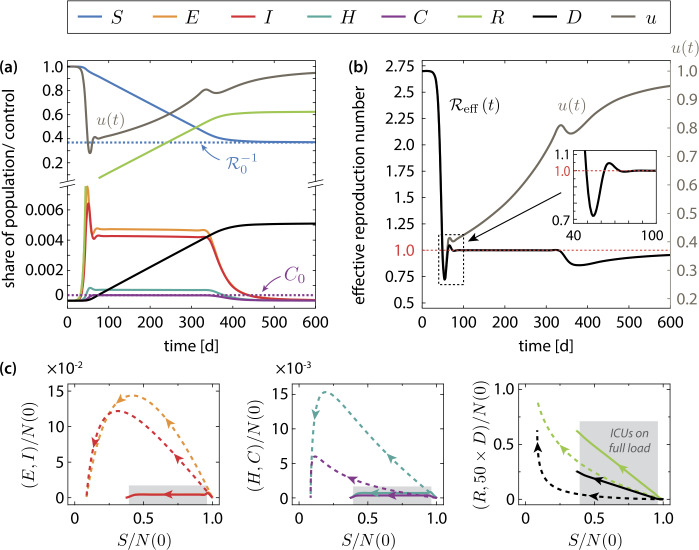
Optimal transmission control for $C_{0}=30{,}000$ available ICUs. (**a**) Temporal evolution of the optimally controlled epidemic. The susceptible population terminates slightly below the critical value $\mathcal{R}_{0}^{-1}$, which guarantees herd immunity and rules out a second wave of the epidemic. Moreover, the optimal control ensures that the available number of ICUs is not exceeded by the critically ill: $C (t )< C_{0}$ for all $t\in [0,\infty )$. A more detailed plot of the ICU load is given in Fig. 5(c). (**b**) Effective reproduction number (6) corresponding to the optimally steered intervention. The optimal mean contact reduction $u (t )$ is shown for comparison. (**c**) Comparison of the trajectories of the uncontrolled (dashed lines) and the optimally controlled epidemic (solid lines) in different projections of the state space. The arrows indicate the direction of time. The grey shaded region highlights the critical period

With optimal control of the transmission rate (in the sense of Sect. 3) via accordingly steered NPIs, the epidemic develops dramatically different from the uncontrolled case. The whole intervention is shown in Fig. 4 and can be structured into three phases:

1. The intervention begins with a strict initial “lockdown” that is built up over a period of about 25 days (starting around day 25), see Fig. 4(a), (b). The effective reproduction number (6) must be held below one $\mathcal{R}_{\text{eff}}<1$ for about 13 days, see Fig. 4(b) and Fig. 5(b). This strict initial intervention breaks the early exponential growth and damps the peak number of infected such that an overshoot of the critically ill population beyond $C_{0}$ is just barely avoided, see Fig. 4(a) and Fig. 5(c).

**Figure 5:**
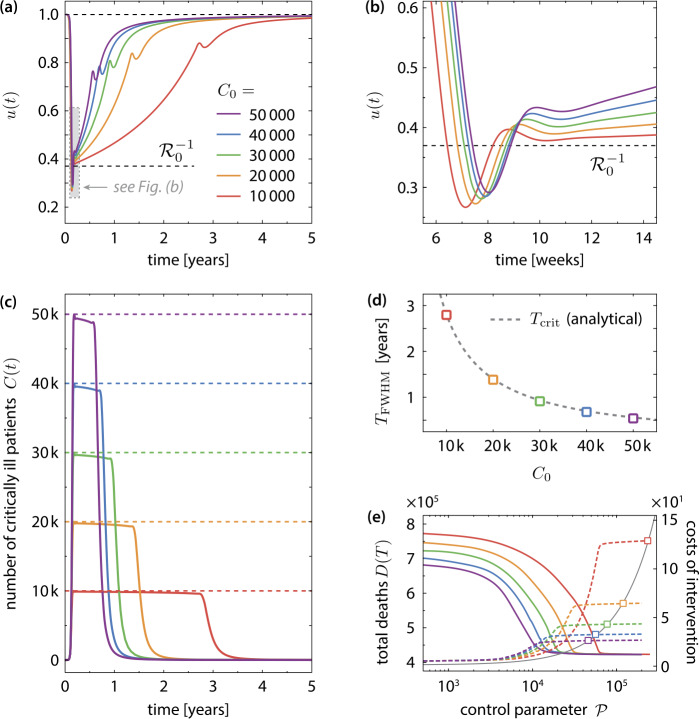
(**a**) Optimal time evolution of the transmission control function $u (t )$ for different values of $C_{0}$. The value of $C_{0}$ is color-coded. In all scenarios, the interventions start with a strict lockdown, where $u (t )$ is reduced below $\mathcal{R}_{0}^{-1}$ for about 10 to 12 days. This initial lockdown is followed by a long “critical period” during which the measures are gradually relaxed. The length of this period is determined by the peak number of simultaneously critically infected $C_{0}$. Towards the end of the intervention, a moderate tightening of the NPIs is required. (**b**) Same as (**a**), but zoomed on the region with $u (t )<\mathcal{R}_{0}^{-1}$. (**c**) By optimal transmission control, the number of patients in a critical state *C* is kept below the limiting value $C_{0}$ at all times. (**d**) Characteristic time span $T_{\text{FWHM}}$ of the critical period during which the peak number of simultaneously infected must be held constant. The dashed line shows the analytical approximation $T_{\text{crit}}$ given in Eq. (17). (**e**) Total number of disease-related deaths (solid lines) and total costs of the measures (dashed lines) at the end of the epidemic vs. the control parameter $\mathcal{P}$ (see Sect. 3). The optimized transmission function minimizes the number of disease-related deaths to a $C_{0}$-independent value for $\mathcal{P}\to \infty $, but to a high cost in the case of low $C_{0}$. The squares indicate the minimal values of $\mathcal{P}$ that guarantee $C(t)< C_{0}$ for all times
2. The initial lockdown is followed by a long period (about 300 days in the case of $C_{0}=30\text{,}000$), which is denoted as the “critical period” in the following, during which the number of simultaneously active cases is kept approximately constant. This corresponds to an effective reproduction number $\mathcal{R}_{\text{eff}}\approx 1$, see Fig. 4(b). During this phase, the intensive care system is constantly stressed by slightly less than $C_{0}$ patients in a critical state. This situation must of course be avoided in reality by all means, in particular, since stochastic fluctuations of the case number are not included in the deterministic model (1a)–(1g) at all. During this period, the NPIs are relaxed on a gradually increasing rate, but initially (when the disease is not yet widespread in the population) only very slowly, see Fig. 4(b). The duration of the critical period scales with $C_{0}^{-1}$. Further details are discussed in Sect. 4.3 below.
3. After the critical period, i.e., when the number of active cases starts to decay, a final moderate tightening of the measures is required. This is reflected by a notable dip in the transmission control function and a reduction of the effective reproduction number below one, see Fig. 4(b). This final intervention reflects the requirement to meet the herd immunity threshold towards the end of the intervention. An unnecessarily wide overshooting into the stable regime would result in additional infections and deaths, see Sect. 4.4. Finally, the measures are lifted on a gradually decreasing rate while the system slowly approaches the herd immunity threshold.

Figure 4(c) shows the trajectories of the controlled and the uncontrolled epidemic in different state space projections. By controlling the transmission of infection, the enormous excursion of the trajectory is prevented and the optimal path to a stable disease-free stationary state is taken. Note that the uncontrolled epidemic terminates far in the stable regime ($S(T)/N (T )\ll \mathcal{R}_{0}^{-1}$), whereas in the optimally controlled case the final state is just slightly below the stability threshold .

We point out that the optimal transmission control described above differs from the results obtained for similar optimization problems considered in Refs. [43, 44, 47], which do not exhibit the distinct structural features of the intervention (initial lockdown, critical period, final phase intervention) presented here. A comparable result was described in Ref. [48], where the intervention was divided into four different phases which essentially coincide with our findings. Merely the lockdown was further subdivided into a “quick activation of a strong lockdown” and a “light lockdown release.”

### Dependence on the maximum number of simultaneously critical cases

The state-dependent mortality rate (4a)–(4b) effectively imposes a state-constraint that strictly enforces $C< C_{0}$ for $\mathcal{P}\to \infty $, i.e., a maximum number of simultaneously infected in a critical condition. In principle, this allows to investigate the optimal control of other (less extreme) scenarios, where the maximum number of simultaneously critically infected should be held far below the number of available ICUs (i.e., the meaning of $C_{0}$ will be reinterpreted). In this case, the increased mortality rate $f_{1}$ is an artificial parameter that penalizes the excess of the critically infected population over a freely chosen threshold of $C_{0}$. By ramping up the control parameter $\mathcal{P}$, an optimal solution with $C (t )< C_{0}$ for all $t\in [0,\infty )$ is found, that is independent of $f_{1}$.

Figure 5 shows the optimal control for different values of $C_{0}$. The time course of the optimally controlled transmission rate is qualitatively the same for all considered values of $C_{0}$, see Fig. 5(a), (b). Most notably, the time scale of the entire intervention scenario is governed by the duration of the critical period, during which the number of critical patients is held at , see Fig. 5(c). We characterize this time scale by the full width half maximum (FWHM) time $T_{\text{FWHM}}=t_{2}-t_{1}$, where $t_{1}$ and $t_{2}>t_{1}$ are the two points in time at which the number of critically infected equals half the allowed maximum value: $C (t_{1} )=C (t_{2} )=C_{0}/2$. As shown in Fig. 5(d), the FWHM time scales inversely with the peak number of simultaneously infected in a critical state: $T_{\text{FWHM}}\sim C_{0}^{-1}$. The minimization of the disease-related deaths is controlled by the parameter $\mathcal{P}$ in the terminal cost function (7b). Figure 5(e) displays the progression of the optimization routine into the targeted optimal state (i.e., without excess of $C_{0}$) while $\mathcal{P}$ is ramped up. At a certain value of $\mathcal{P}$, which depends on $C_{0}$, the routine reaches a plateau where both the number of disease-related deaths as well as the total costs of the intervention measures $\int _{0}^{T}\mathrm{d}t \,\mathcal{C} (u (t ) )$ become constant. The corresponding values of $\mathcal{P}$, which correspond to the scenario that fully avoids excess of critically ill over $C_{0}$, are located on that plateau and are marked by square symbols in Fig. 5(e). The optimized transmission function minimizes the number of disease-related deaths to a $C_{0}$-independent value $D_{\text{min}} (T )$ for $\mathcal{P}\to \infty $, but at total cost that scales with $C_{0}^{-1}$. An analytical estimate of the minimum attainable number of deaths is given in Eq. (18).

Within the present model, further reduction of disease-related deaths below $D_{\text{min}} (T )$ can only be achieved by pharmaceutical interventions, in particular by vaccination. The result of the $C_{0}$-independent number of deaths at the end of the epidemic is an artifact of the simplified modeling framework, in which a homogeneous population with an averaged set of parameters is considered. Since the mortality rate typically strongly depends on age and health condition, it might be advisable to extend the model and divide the compartments into several age or risk groups as in Refs. [11, 45, 54, 61]. The so-extended model features a matrix-valued transmission rate, which describes the infections caused by contacts within and between different groups, that could be further optimized by intra- and intergroup-specific measures. This is, however, beyond the scope of this paper.

### Analysis of the critical period

The numerical results shown in Fig. 4(a), (b) indicate that during the critical period the populations *S*, *R*, and *D* change approximately linear, while the active cases (*E*, *I*, *H*, *C*) are practically constant. To gain further insights, we consider the ansatz (for $t>t^{*}$)

$$\begin{aligned} S (t ) \approx N (0 )-\gamma _{S} \bigl(t-t^{\ast } \bigr),\qquad R (t ) \approx \gamma _{R} \bigl(t-t^{\ast } \bigr), \qquad D (t ) \approx \gamma _{D} \bigl(t-t^{\ast } \bigr), \end{aligned}$$

where $t^{\ast }$ is a reference time that depends on the initial conditions, $\gamma _{S}$, $\gamma _{R}$, $\gamma _{D}$ are initially unknown rates and the infected sub-populations $(E,I,H,C )\approx (E^{\ast },I^{\ast },H^{\ast },C_{0} )$ are constant. From substituting the ansatz into the model equations (1a)–(1g), one obtains by a straightforward calculation analytical expressions for the rates

$$\begin{aligned} \gamma _{S} =\frac{1-c (1-f_{0} )}{ (1-m )c} \gamma _{c}C_{0},\qquad \gamma _{R} = \frac{1-c (1-mf_{0} )}{ (1-m )c}\gamma _{c}C_{0},\qquad \gamma _{D} =f_{0}\gamma _{c}C_{0}, \end{aligned}$$

and the constants

$$\begin{aligned} E^{\ast } \approx \frac{1}{\gamma _{l}}\gamma _{S}, \qquad I^{\ast } \approx \frac{1}{\gamma _{i}}\gamma _{S}, \qquad H^{*} \approx \frac{1}{\gamma _{h}}\frac{1}{cf_{0}}\gamma _{D}. \end{aligned}$$

The rate of new infections per day $\gamma _{S}$ during the critical period depends only on the parameters of the disease and the maximum capacity $C_{0}$. Note that it holds $\gamma _{S}=\gamma _{R}+\gamma _{D}$, i.e., the number of active cases remains constant since susceptibles become infected at the same rate on which active cases either recover or die. The number of active cases in this *dynamical equilibrium* is a multiple of $C_{0}$:

$$\begin{aligned} N_{\text{act}}^{\ast }=E^{\ast }+I^{*}+H^{\ast }+C^{\ast } = \biggl( \frac{1-c (1-f_{0} )}{c (1-m )} \biggl( \frac{1}{\gamma _{l}}+ \frac{1}{\gamma _{i}} \biggr)\gamma _{c}+ \frac{1}{c} \frac{\gamma _{c}}{\gamma _{h}}+1 \biggr)C_{0}. \end{aligned}$$

With the parameters listed in Table 1, we find $N_{\text{act}}^{\ast }\approx 28.3 C_{0}$, i.e., one out of about thirty infections turns critical.

Let us now come to the major results of this section. The ansatz stated above yields an instantaneous relationship between the current value of the transmission control function and the share of the susceptibles on the total population $S(t)/N(t)$, which is

13 $$ u (t )\approx \frac{1}{\mathcal{R}_{0}} \frac{N (t )}{S (t )}\approx \frac{1}{\mathcal{R}_{0}} \biggl(1- \frac{\gamma _{S}}{N (0 )} \bigl(t-t^{\ast } \bigr) \biggr)^{-1}= \biggl( \mathcal{R}_{0}- ( \mathcal{R}_{0}-1 ) \frac{t-t^{\ast }}{T_{\text{crit}}} \biggr)^{-1} $$

for a certain range of *t* in $t^{\ast }< t< T_{\text{crit}}$ with $T_{\text{crit}}$ defined below. Here, we approximated $N (t )\approx N (0 )$ (since $\gamma _{D}\ll \gamma _{S}$). Note that Eq. (13) implies $\mathcal{R}_{\text{eff}}\approx 1$ during the critical period. This approximate relation is an interesting result, as it hints that the obtained optimal control steers the system’s trajectory close to the stability boundary. Comparison with the stability criterion for the disease-free stationary state $\mathcal{R}_{0}<\bar{N}/\bar{S}$, see Eq. (19), suggests that during the critical period one must make sure that $\mathcal{R}_{\text{eff}} (t )<1$, i.e.,

14 $$ u (t )< \frac{1}{\mathcal{R}_{0}} \frac{N (t )}{S (t )}. $$

**Figure 6:**
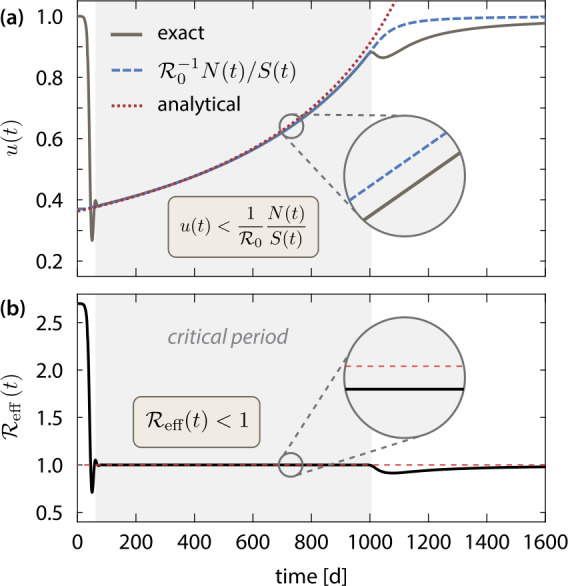
(**a**) Analysis of the optimal mean contact reduction $u (t )$ during the critical period, where the number of simultaneously infected must be kept constant (the plot is for $C_{0}=10\text{,}000$). The numerically exact result is plotted along with the stability boundary $\mathcal{R}_{0}^{-1}N (t )/S (t )$ (blue dashed line) and the analytical approximation (13) (red dotted line). The inset shows that the optimal control respects the stability requirement (14) during the critical period. (**b**) Plot of effective reproduction number $\mathcal{R}_{\text{eff}} (t )$ corresponding to the optimal control. Throughout the critical period, $\mathcal{R}_{\text{eff}} (t )$ is kept slightly below one

This allows to have a stable control of the number of active cases, while the intervention measures can be gradually relaxed. Stable means that sufficiently small fluctuations of the number of infected are damped and do not lead to a new exponential outbreak of the epidemic. Indeed, substituting $u (t )= (1+\varepsilon )N (t )/( \mathcal{R}_{0}S (t ))$ into the model equations (1a)–(1g) yields a linear, autonomous dynamical system (up to the state-dependent mortality rate (4a)–(4b)), which is easily seen to evolve close to a stable dynamical equilibrium for $\varepsilon <0$ and $\vert \varepsilon \vert \ll 1$, see Appendix B. The optimal transmission control function is shown in Fig. 6 along with the analytical approximation (13), the stability criterion (14) and the corresponding effective reproduction number for the critical period.

We formulate the stability criterion (14) once again in a different way. Since it holds $S (t )\approx N (0 )-N_{\text{cases}} (t )$, where $N_{\text{cases}} (t )$ is the cumulative number of cases that includes next to the active cases also the recovered and deceased population $N_{\text{cases}} (t )=N_{\text{act}} (t )+R (t )+D (t )$, the stability criterion (14) can be written as

15 $$ u (t )< \frac{1}{\mathcal{R}_{0}} \biggl(1- \frac{N_{\text{cases}} (t )}{N (0 )} \biggr)^{-1}. $$

Hence, since the optimal control depends solely on the cumulative number of cases, it is crucial to have an accurate estimate of $N_{\text{cases}}$ at any time during the critical period. Next, we derive an upper limit for the admissible rate of change of $u(t)$. By differentiating Eq. (14), using Eq. (1a) and approximating $N (t )\approx N (0 )$ as well as $I(t)\approx I^{\ast }$ (see above), we obtain

$$ \dot{u} (t )< \frac{1}{\mathcal{R}_{0}} \biggl(- \frac{\dot{D} (t )}{S (t )}- \frac{N (t )}{S^{2} (t )} \dot{S} (t ) \biggr)< \frac{N (t )}{S (t )}u (t ) \frac{\gamma _{i}I (t )}{N (t )}< \biggl( \frac{N (t )}{\mathcal{R}_{0}S (t )} \biggr)^{2} \frac{\mathcal{R}_{0}\gamma _{S}}{N (0 )}. $$

Using the approximation (13), the rate on which the measures can be relaxed is limited by the square of the current value of the control function. It holds

16 $$ \dot{u} (t )< \frac{\mathcal{R}_{0}\gamma _{S}}{N (0 )}u^{2} (t ). $$

The numerically computed optimal control obeys the criteria (15)–(16), see Fig. 6, and is therefore (weakly) stable against small perturbations. The merely weak stability reflects the demand for minimal socio-economic costs, see Sect. 3. The two rules (15)–(16) for the optimal and stable steering of the transmission control function are widely independent of the details of the current model system. Equivalent results for a stable dynamical equilibrium with a constant number of infected cases are easily obtained for the much simpler SIR model.

The characteristic duration $T_{\text{crit}}$ of the critical period is estimated from Eq. (13) and the condition $u (t^{\ast }+T_{\text{crit}} )\approx 1$. One obtains

17 $$ T_{\text{crit}}\approx \frac{N (0 )}{\gamma _{S}} \biggl(1- \frac{1}{\mathcal{R}_{0}} \biggr) \propto \frac{N (0 )}{C_{0}} \biggl(1-\frac{1}{\mathcal{R}_{0}} \biggr), $$

which is in excellent agreement with the numerically obtained values for the FWHM time plotted in Fig. 5(d). Finally, we estimate of the total number of disease-related deaths from $D (T )\approx D (t^{*}+T_{\text{crit}} ) \approx \gamma _{D}T_{\text{crit}}$ as

18 $$\begin{aligned} D (T ) & \approx N (0 ) \frac{\gamma _{D}}{\gamma _{S}} \biggl(1-\frac{1}{\mathcal{R}_{0}} \biggr)=N (0 ) \frac{ (1-m )cf_{0}}{1-c (1-f_{0} )} \biggl(1- \frac{1}{\mathcal{R}_{0}} \biggr), \end{aligned}$$

which is independent of $C_{0}$, cf. Sect. 4.2 and Fig. 5(e).

### Final phase of the intervention

**Figure 7:**
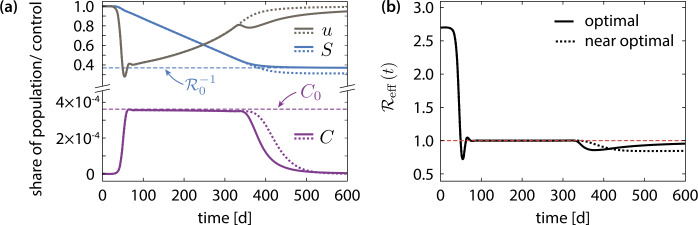
(**a**) Comparison of the optimal (dashed) and near optimal (dotted) control of the mean contact reduction. In the near optimal control, the strengthening of the measures in the final phase of the intervention is omitted. Instead, the near optimal control adheres to the stability boundary (14) and causes an overshoot of the susceptible population below the stability threshold ($S (T )< N (T )/\mathcal{R}_{0}$), (**b**) Plot of the corresponding effective reproduction number

Finally, we briefly discuss the moderate tightening of the measures in the last (third) phase of the intervention. To this end, we compare the optimal intervention scenario with a nearly optimal control, which lacks the last intervention phase as shown in Fig. 7. In the case of nearly optimal control, the mean contact reduction after the initial lockdown continuously follows the course of the stability boundary (14), which leads to an excess of infections beyond the required herd immunity threshold, see Fig. 7(a). The final state therefore is considerably further in the stable region than required. This implies that more infections than necessary are passed through, which results in exceeding the minimum number of deaths (not shown), cf. Eq. (18). In order to prevent this, the measures must be slightly tightened towards the end of the intervention such that the number of active cases is diminished and thus an unnecessary decrease of the susceptible population below the herd immunity threshold is avoided.

## Summary and conclusions

Non-pharmaceutical measures to control the spread of infectious diseases and to prevent a potential collapse of the health care system must be precisely coordinated in terms of timing and intensity. Based on well-calibrated mathematical models, the optimal intervention strategy for specific scenarios and objectives can be computed using continuous-time optimal control theory.

In this paper, an extended SEIR model was calibrated to reproduce the data of the initial exponential growth phase of the COVID-19 pandemic in Germany. Optimal control theory has been applied for the scenario in which an effective vaccine is impossible or will never be found and the epidemic must be controlled with purely non-pharmaceutical measures. We have computed the optimal control of the transmission rate that satisfies competing objectives: First, the minimization of the disease-related deaths by strictly avoiding an overflow of intensive care resources and, second, the suppression of a second outbreak by establishing sufficient natural immunity at the end of the measures. Moreover, the total costs of the intervention shall be kept at a necessary minimum for socio-economic reasons.

The optimal control obtained in this paper is a single-intervention scenario that exhibits several notable features, which allow to structure the whole intervention into three distinct phases: (i) strict initial lockdown, (ii) critical period and (iii) moderate tightening of measures in the final phase. The obtained control differs from the results described in related works [43, 44, 47], but is comparable to the NPI strategy presented in Ref. [48]. We have shown that our optimized time-resolved NPI policy is robust under parameter variation and developed a qualitative understanding of its distinct phases.

The comparison of the computed optimal transmission control function with the stability criteria (15)–(16) reveals, however, that the obtained solution is in fact a tightrope walk close to the stability boundary of the system, where socio-economic costs and the risk of a new outbreak must be constantly balanced against one another. Furthermore, our analysis clearly shows that the goal of achieving herd immunity via natural infections is either extremely expensive (in terms of socio-economic costs due to measures maintained over a long period of time) or extremely dangerous (due to the constantly high load on intensive care resources just below the stability limit). Note that the values of $C_{0}$ considered in the computations are relatively high throughout. In any case, in view of the long duration and the enormous number of infections that this route entails, as well as the uncertain role of sequelae and the uncertain prospects for appropriate vaccines, it is strongly advisable to consider other strategies, in particular the attempt to reduce the number of cases to a level that is manageable for case tracking [62] or to eradicate the epidemic completely [63].

## Figures and Tables

**Figure 8:**
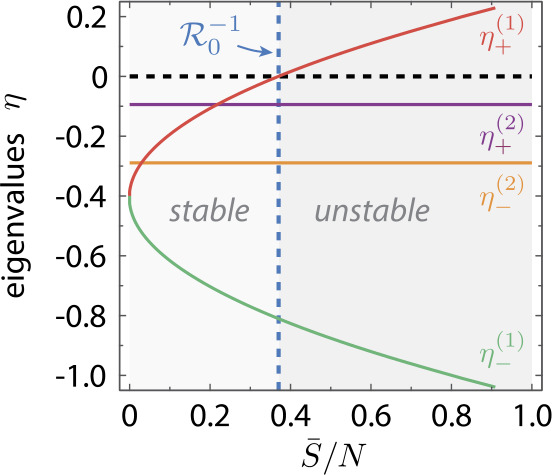
The stability of the disease-free stationary state depends on the size of the susceptible population *S̄* and the basic reproduction number $\mathcal{R}_{0}$. For $\bar{S}/N<\mathcal{R}_{0}^{-1}$, the outbreak of an epidemic is suppressed by a sufficiently high degree of herd immunity

**Figure 9:**
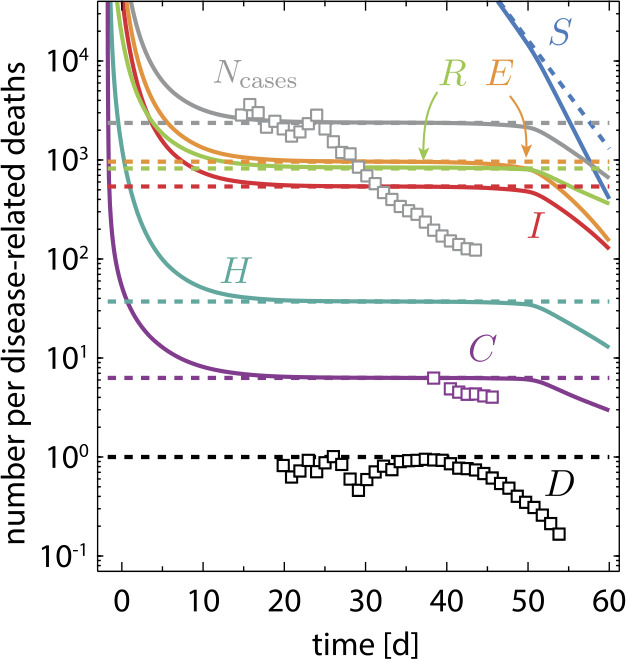
Ratio of the state variables in the initial exponential growth phase and the number of disease related deaths. The numerically exact solution (solid lines) is plotted along with an analytical approximation (solid lines) that holds in the early stage of the epidemic. The corresponding algebraic relations are used to describe ratios between different sub-populations to facilitate the parameter adjustment. Symbols indicate the reported number of disease-related deaths (black), estimated number of cases (grey) and estimated ICU load (purple) of the COVID-19 pandemic in Germany. See the text for details. During mid-March, strict social distancing measures were implemented, that flattened the initial exponential growth

## Appendix A: Stability analysis of the disease-free stationary state

Without intervention, i.e. $u=1$, the system (1a)–(1g) has a family of disease-free stationary states $\bar{\mathbf{x}}= (\bar{S},0,0,0,0,\bar{R},\bar{D} )$. The stability of a stationary state with respect to small perturbations $\bar{\mathbf{x}}\to \bar{\mathbf{x}}+\delta \mathbf{x} (t )$ is determined by the sign of the real parts of the eigenvalues *η* of the linearized system’s coefficient matrix

$$ A (\bar{\mathbf{x}} )= \begin{pmatrix} 0 & 0 & -\beta \bar{S}/\bar{N} & 0 & 0 & 0 & 0 \\ 0 & -\gamma _{l} & \beta \bar{S}/\bar{N} & 0 & 0 & 0 & 0 \\ 0 & \gamma _{l} & -\gamma _{i} & 0 & 0 & 0 & 0 \\ 0 & 0 & (1-m )\gamma _{i} & -\gamma _{h} & (1-f_{0} )\gamma _{c} & 0 & 0 \\ 0 & 0 & 0 & c\gamma _{h} & -\gamma _{c} & 0 & 0 \\ 0 & 0 & m\gamma _{i} & (1-c )\gamma _{h} & 0 & 0 & 0 \\ 0 & 0 & 0 & 0 & f_{0}\gamma _{c} & 0 & 0 \end{pmatrix}. $$

with $\bar{N}=\bar{S}+\bar{R}$. From the characteristic polynomial

$$ 0=\chi (\eta )=\det \bigl(A (\bar{\mathbf{x}} )-\eta I \bigr), $$

one obtains the eigenvalues

$$\begin{aligned} &\eta _{\pm }^{ (1 )} =\frac{1}{2} \bigl(- (\gamma _{i}+ \gamma _{l} )\pm \sqrt{ (\gamma _{i}- \gamma _{l} )^{2}+4 \mathcal{R}_{0}\gamma _{i}\gamma _{l} \bar{S}/\bar{N}} \bigr), \\ &\eta _{\pm }^{ (2 )} =\frac{1}{2} \bigl(- (\gamma _{c}+ \gamma _{h} )\pm \sqrt{ (\gamma _{c}- \gamma _{h} )^{2}+4c (1-f_{0} )\gamma _{c}\gamma _{h}} \bigr), \end{aligned}$$

and the threefold degenerate eigenvalue $\eta ^{ (0 )}=0$. Since $c (1-f_{0} )<1$, it holds $\eta _{\pm }^{ (2 )}<0$. The leading eigenvalue is $\eta _{+}^{ (1 )}$, which is negative for

19 $$ \bar{S}/\bar{N}< \mathcal{R}_{0}^{-1}, $$

see Fig. 8. Hence, the disease-free stationary state is unstable if the susceptible population size exceeds a critical threshold value that is given by the inverse basic reproduction number (5). For $\bar{S}/\bar{N}<\mathcal{R}_{0}^{-1}$ an epidemic outbreak is suppressed by a sufficiently high degree of herd immunity.

## Appendix B: Dynamical equilibrium and stability during the critical period

For the stability analysis of the dynamical equilibrium during the critical period it is sufficient to consider the $(S,E,I )$-block of the system (1a)–(1g), which drives the remaining equations. At first,

$$\begin{aligned} \dot{S} =-\beta u (t )\frac{IS}{N}, \qquad \dot{E} =\beta u (t ) \frac{IS}{N}-\gamma _{l}E, \qquad \dot{I} =\gamma _{l}E- \gamma _{i}I, \end{aligned}$$

is a nonlinear and non-autonomous dynamical system. Substituting the control function

$$ u (t )= (1+\varepsilon ) \frac{1}{\mathcal{R}_{0}}\frac{N (t )}{S (t )}, $$

yields a linear and autonomous system

$$\begin{aligned} \dot{S} =- (1+\varepsilon )\gamma _{i}I, \qquad \dot{E} = (1+ \varepsilon )\gamma _{i}I-\gamma _{l}E, \qquad \dot{I} = \gamma _{l}E-\gamma _{i}I. \end{aligned}$$

For $\varepsilon =0$, it is easily seen that $\dot{E}+\dot{I}=0$, such that there exists a dynamical equilibrium with a constant number of actively infected: $E^{*}+I^{*}=\text{const}$., where $E^{*}= (1+\gamma _{i}/\gamma _{l} )I^{*}$. The corresponding susceptible population is linearly decreasing on a rate $\gamma _{S}=\gamma _{i}I^{*}$. The stability of the dynamical equilibrium $(E^{*},I^{*} )$ is determined by the roots of the characteristic polynomial

$$ 0=\varLambda ^{2}+ (\gamma _{l}+\gamma _{i} ) \varLambda -\gamma _{l} \gamma _{i}\varepsilon $$

that are obtained as

$$ \varLambda _{\pm }=-\frac{\gamma _{l}+\gamma _{i}}{2}\pm \sqrt{ \biggl( \frac{\gamma _{l}+\gamma _{i}}{2} \biggr)^{2}+\gamma _{l}\gamma _{i} \varepsilon }. $$

Clearly, for $\varepsilon >0$, the dynamical equilibrium becomes unstable due to $\varLambda _{+}>0$. The stability boundary is given by $\varepsilon =0$, on which the dynamical equilibrium exists. The optimal control obtained in the main text drives the system slightly below the stability boundary ($\varepsilon <0$, $\vert \varepsilon \vert \ll 1$), see Fig. 6(a). In this case it holds $\varLambda _{\pm }<0$, such that the system is weakly stable against small perturbations, because the number of active cases is constantly decreasing.

## Appendix C: Parameter adjustment

The parameters are adjusted such that the model reproduces the data of the early exponential growth phase of the COVID-19 pandemic in Germany. It is of course questionable to calibrate an epidemic model to a single country, but in a scenario with extensive border closures this seems to be justified. In the exponential growth phase of the epidemic, all sub-populations grow exponentially with the same rate, see Fig. 2(b). This observation can be exploited to derive a series of algebraic equations (which hold approximately in the initial phase of the epidemic) that relate all state variables to each other. On the basis of empirical data (reported number of cases and deaths etc.), several missing model parameters can be directly determined from the algebraic relations. The number of reported cases and deaths used in this study is based on the figures provided by the Robert Koch-Institute [64, 65].

One starts with the ansatz

20 $$\begin{aligned} I (t ) \approx I (0 )\mathrm{e}^{\varGamma t}, \qquad S (t ) \approx N (0 ), \end{aligned}$$

where *Γ* is the initial exponential growth rate that is estimated from reported data (see Fig. 2(b)) as $\varGamma \approx 0.26 \text{d}^{-1}$ (doubling time of infections within $\varGamma ^{-1}\log { (2 )}\approx 2.67 \text{d}$). Substituting Eq. (20) in Eqs. (1b)–(1c) yields

$$ E (t )\approx \frac{1}{\gamma _{l}} (\varGamma +\gamma _{i} )I (t ) $$

and the relation between the growth rate and $\mathcal{R}_{0}$:

21 $$ \biggl(1+\frac{\varGamma }{\gamma _{l}} \biggr) \biggl(1+ \frac{\varGamma }{\gamma _{i}} \biggr)= \mathcal{R}_{0}. $$

Note that Eq. (21) is equivalent to the equation for the leading eigenvalue $\eta _{+}^{ (1 )}$ if the whole population is susceptible, i.e. $\varGamma =\eta _{+}^{ (1 )}\vert _{\bar{S}=N(0)}$ (see Appendix A). Hence, Eq. (21) implies that the exponential growth rate *Γ* changes sign at $\mathcal{R}_{0}=1$, i.e., the epidemic recedes for $\mathcal{R}_{0}<1$. The mean incubation period was reported to be 5.1d, but there are indications that the latency time may be shorter [66]. Assuming the onset of infectiousness 2.5d before the onset of symptoms, this implies an average latency period of $\gamma _{l}^{-1}=2.6 \text{d}$, i.e., the latency period is assumed to equal roughly half of the incubation period. The reported values of the basic reproduction number $\mathcal{R}_{0}$ are heavily scattered. According to the Robert Koch Institute, serious estimates range between 2.4 and 3.3 [67]. In the following $\mathcal{R}_{0}=2.7$ shall be used, which is situated approximately in the middle of the interval in question. From Eq. (21), the corresponding average infectious period is obtained as $\gamma _{i}^{-1}\approx 2.35 \text{d}$.

The overall infection fatality rate of COVID-19 was estimated as 0.66% [68], such that $(1-m )cf_{0}=0.0066$. On April 8, the Robert Koch Institute reported that a fraction of $f_{0}=0.31$ patients in a critical state died (without ICU overflow) [69]. Finally, the fraction of infected with at most mild symptoms is estimated as $m=0.92$, such that $c=0.0213/ (1-m )\approx 0.266$.

Substituting the exponential ansatz (20) in Eqs. (1d)–(1g), yields

$$\begin{aligned} &H (t ) \approx \frac{ (1-m )}{K} \frac{\gamma _{i}}{\varGamma } \biggl(1+ \frac{\gamma _{c}}{\varGamma } \biggr)I (t ),\qquad C (t ) \approx \frac{ (1-m )c}{K} \frac{\gamma _{i}\gamma _{h}}{\varGamma ^{2}}I (t ), \\ &R (t ) \approx \frac{\gamma _{i}}{\varGamma } \biggl(m+ \frac{ (1-m ) (1-c )}{K} \frac{\gamma _{h}}{\varGamma } \biggl(1+\frac{\gamma _{c}}{\varGamma } \biggr) \biggr)I (t ),\qquad D (t ) \approx \frac{ (1-m )cf_{0}}{K} \frac{\gamma _{i}\gamma _{c}\gamma _{h}}{\varGamma ^{3}}I (t ) \end{aligned}$$

with $K=1+ (\gamma _{h}+\gamma _{c} )/\varGamma +\gamma _{c} \gamma _{h} (1- (1-f_{0} )c )/\varGamma ^{2}$. The analytically obtained ratio between all sub-population and deaths (which are believed to be the most reliably reported data) are plotted along with the corresponding numerically exact result for the initial uncontrolled epidemic in Fig. 9(b). The analytical results imply the relation

22 $$ D (t )/C (t )=\gamma _{c}f_{0}/\varGamma. $$

Unfortunately, there is only little data available on the demand for ICUs in the early phase of the epidemic. In mid-March 2020, i.e. near the end of the initial exponential growth phase, the German Interdisciplinary Association for Intensive Care and Emergency Medicine (DIVI) initiated a register that reports on the availability of ICUs in Germany [70]. On March 27, 687 out of 1160 hospitals with ICUs contributed to the register and reported a total number of 939 COVID-19 patients in a critical state receiving intensive care [71]. At the same day, 253 disease-related deaths were reported. From the estimated ratio $C/D\approx 6.3$ (the actual number of critical patients was estimated based on the ratio of contributing and non-contributing hospitals as $C\approx 1586$), the average period after which patients in a critical state either recover or die, is estimated from Eq. (22) as $\gamma _{c}^{-1}\approx 7.5 \text{d}$.

Finally, assuming that only $r=2/3$ of all cases have been discovered initially and an assumed average time delay between infection and report of cases of $\Delta t_{r}=5 \text{d}$, the number of actual cases is estimated from the number of reported cases as $N_{\text{cases}}^{\text{est}} (t )=r^{-1}N_{\text{cases}}^{ \text{rep}} (t+\Delta t_{r} )=r^{-1}\mathrm{e}^{\varGamma \Delta t_{r}}N_{\text{cases}}^{\text{rep}} (t )\approx 5.5 N_{ \text{cases}}^{\text{rep}} (t )$. This yields a good agreement between the simulated number of cases ($N_{\text{cases}}=E+I+H+C+R+D$) and $N_{\text{cases}}^{\text{est}}$ before measures came into force, see Fig. 2(a) and Fig. 9. The average time between infection and death $\Delta t_{d}$ can be estimated from the ratio $N_{\text{cases}}^{\text{est}} (t )/D (t ) \approx 2370$ (see Fig. 8(b)) and $N_{\text{cases}}^{\text{est}} (t-\Delta t_{d} )=N_{ \text{cases}}^{\text{est}} (t )\mathrm{e}^{-\varGamma \Delta t_{d}}=D (t )$ as $\Delta t_{d}\approx 29.9 \text{d}$.

## Appendix D: Two-point boundary value problem

Rescaling the populations $\mathbf{x}= (S,E,I,H,C,R,D )$ subject to the dynamical system (1a)–(1g) by the initial population size $N(0)$ and using $N (t )=N (0 )-D (t )$, see Eq. (3), we obtain the equations of motion for the rescaled sub-populations $\tilde{\boldsymbol{x}} (t )=\mathbf{x} (t )/N (0 )$ as

23a $$\begin{aligned} &\dot{\tilde{S}} =-\beta u (t ) \frac{\tilde{I}\tilde{S}}{1-\tilde{D}}, \end{aligned}$$

23b $$\begin{aligned} &\dot{\tilde{E}} =\beta u (t ) \frac{\tilde{I}\tilde{S}}{1-\tilde{D}}-\gamma _{l} \tilde{E}, \end{aligned}$$

23c $$\begin{aligned} &\dot{\tilde{I}} =\gamma _{l}\tilde{E}-\gamma _{i} \tilde{I}, \end{aligned}$$

23d $$\begin{aligned} &\dot{\tilde{H}} = (1-m )\gamma _{i}\tilde{I}+ \bigl(1-f( \tilde{C}/ \tilde{C}_{0}) \bigr)\gamma _{c}\tilde{C}- \gamma _{h} \tilde{H}, \end{aligned}$$

23e $$\begin{aligned} &\dot{\tilde{C}} =c\gamma _{h}\tilde{H}-\gamma _{c} \tilde{C}, \end{aligned}$$

23f $$\begin{aligned} &\dot{\tilde{R}} =m\gamma _{i}\tilde{I}+ (1-c )\gamma _{h} \tilde{H}, \end{aligned}$$

23g $$\begin{aligned} &\dot{\tilde{D}} =f(\tilde{C}/\tilde{C}_{0})\gamma _{c} \tilde{C}, \end{aligned}$$

where $\tilde{C}_{0}=C_{0}/N(0)$. The co-state equations of the optimal control problem considered in Sect. 3 for the rescaled Lagrange multipliers $\tilde{\boldsymbol{\lambda }} (t )=N (0 ) \boldsymbol{\lambda } (t )$ read

$$\begin{aligned} &\dot{\tilde{\lambda }}_{S} (t ) =L+\beta ( \tilde{\lambda }_{S}-\tilde{\lambda }_{E}) u \frac{\tilde{I}}{1-\tilde{D}}, \\ &\dot{\tilde{\lambda }}_{E} (t ) =L+\gamma _{l} ( \tilde{ \lambda }_{E}-\tilde{\lambda }_{I}), \\ &\dot{\tilde{\lambda }}_{I} (t ) =L+\beta ( \tilde{\lambda }_{S}-\tilde{\lambda }_{E}) u \frac{\tilde{S}}{1-\tilde{D}}+ \gamma _{i} \bigl(\tilde{\lambda }_{I}- \tilde{\lambda }_{H}+m (\tilde{\lambda }_{H}-\tilde{\lambda }_{R}) \bigr), \\ &\dot{\tilde{\lambda }}_{H} (t ) =L+\gamma _{h} \bigl( \tilde{\lambda }_{H}-\tilde{\lambda }_{R}+c ( \tilde{\lambda }_{R}- \tilde{\lambda }_{C}) \bigr), \\ &\dot{\tilde{\lambda }}_{C} (t ) =L+\gamma _{c} ( \tilde{ \lambda }_{C}-\tilde{\lambda }_{H})+\gamma _{c} \biggl(f \biggl( \frac{\tilde{C}}{\tilde{C}_{0}} \biggr)+ \frac{\tilde{C}}{\tilde{C}_{0}}f' \biggl( \frac{\tilde{C}}{\tilde{C}_{0}} \biggr) \biggr) ( \tilde{\lambda }_{H}- \tilde{\lambda }_{D}), \\ &\dot{\tilde{\lambda }}_{R} (t ) =L, \\ &\dot{\tilde{\lambda }}_{D} (t ) =0, \end{aligned}$$

with

$$ L=-\frac{u\log (u )}{1-\tilde{D}}, \qquad u=\exp { \biggl(\beta (\tilde{\lambda }_{S}- \tilde{\lambda }_{E}) \frac{\tilde{I}\tilde{S}}{1-\tilde{D}} \biggr)}. $$

The initial conditions are taken as

$$\begin{aligned} \tilde{S} (0 ) =1-\tilde{E} (0 ),\qquad \tilde{E} (0 ) =2.41\times 10^{-7},\qquad \tilde{I} (0 ) = \tilde{H} (0 )=\tilde{C} (0 )=\tilde{R} (0 )=\tilde{D} (0 )=0, \end{aligned}$$

and the final time conditions (12) read

$$\begin{aligned} &\tilde{\lambda }_{S} (T ) =-\frac{1}{\varepsilon } \frac{\mathcal{R}_{0}}{1-\tilde{D} (T )} \biggl(1- \frac{\tilde{S} (T )}{1-\tilde{D} (T )} \biggr) \log \biggl( \frac{1}{\varepsilon } \biggl[1- \frac{\mathcal{R}_{0}\tilde{S} (T )}{1-\tilde{D} (T )} \biggr] \biggr), \\ &\tilde{\lambda }_{E,I,H,C,R} (T ) =\frac{1}{\varepsilon } \frac{\mathcal{R}_{0}}{1-\tilde{D} (T )} \frac{\tilde{S} (T )}{1-\tilde{D} (T )}\log \biggl(\frac{1}{\varepsilon } \biggl[1- \frac{\mathcal{R}_{0}\tilde{S} (T )}{1-\tilde{D} (T )} \biggr] \biggr), \\ &\tilde{\lambda }_{D} (T ) =N (0 )\mathcal{P}. \end{aligned}$$

The choice of the initial time conditions guarantees $u (0 )=1$ (no intervention) at the beginning of the scenario.

## Abbreviations

COVID-19: coronavirus disease 2019
FWHM: full width half maximum
ICU: intensive care unit
NPI: non-pharmaceutical intervention
S(E)IR: susceptible-(exposed)-infectious-recovered
